# Exploration and verification a 13-gene diagnostic framework for ulcerative colitis across multiple platforms via machine learning algorithms

**DOI:** 10.1038/s41598-024-65481-8

**Published:** 2024-07-01

**Authors:** Jing Wang, Lin Li, Pingbo Chen, Chiyi He, Xiaoping Niu

**Affiliations:** 1 Department of Gastroenterology, The First Affiliated Hospital of Wan-Nan Medical College, Wuhu, 241001 China; 2 Department of Joint-Orthopedics, The First Affiliated Hospital of Wan-Nan Medical College, Wuhu, 241001 China

**Keywords:** Ulcerative colitis, Diagnostic model, Machine learning algorithms, Gene set enrichment analysis, Immunocytes infiltration, Predictive medicine, Computational biology and bioinformatics, Medical research

## Abstract

Ulcerative colitis (UC) is a chronic inflammatory bowel disease with intricate pathogenesis and varied presentation. Accurate diagnostic tools are imperative to detect and manage UC. This study sought to construct a robust diagnostic model using gene expression profiles and to identify key genes that differentiate UC patients from healthy controls. Gene expression profiles from eight cohorts, encompassing a total of 335 UC patients and 129 healthy controls, were analyzed. A total of 7530 gene sets were computed using the GSEA method. Subsequent batch correction, PCA plots, and intersection analysis identified crucial pathways and genes. Machine learning, incorporating 101 algorithm combinations, was employed to develop diagnostic models. Verification was done using four external cohorts, adding depth to the sample repertoire. Evaluation of immune cell infiltration was undertaken through single-sample GSEA. All statistical analyses were conducted using R (Version: 4.2.2), with significance set at a P value below 0.05. Employing the GSEA method, 7530 gene sets were computed. From this, 19 intersecting pathways were discerned to be consistently upregulated across all cohorts, which pertained to cell adhesion, development, metabolism, immune response, and protein regulation. This corresponded to 83 unique genes. Machine learning insights culminated in the LASSO regression model, which outperformed others with an average AUC of 0.942. This model's efficacy was further ratified across four external cohorts, with AUC values ranging from 0.694 to 0.873 and significant Kappa statistics indicating its predictive accuracy. The LASSO logistic regression model highlighted 13 genes, with LCN2, ASS1, and IRAK3 emerging as pivotal. Notably, LCN2 showcased significantly heightened expression in active UC patients compared to both non-active patients and healthy controls (P < 0.05). Investigations into the correlation between these genes and immune cell infiltration in UC highlighted activated dendritic cells, with statistically significant positive correlations noted for LCN2 and IRAK3 across multiple datasets. Through comprehensive gene expression analysis and machine learning, a potent LASSO-based diagnostic model for UC was developed. Genes such as LCN2, ASS1, and IRAK3 hold potential as both diagnostic markers and therapeutic targets, offering a promising direction for future UC research and clinical application.

## Introduction

Ulcerative colitis (UC) is indeed an inflammatory bowel disease (IBD) that predominantly impacts the mucosal and submucosal layers of the colon and rectum, manifesting as a chronic condition characterized by inflammation and the formation of ulcers in the lining of the colon and rectum. Simultaneously, prolonged UC results in structural damage, amplifying the susceptibility to conditions such as colon cancer and extraintestinal malignancies^1,2^. However, the pathogenesis of UC remains a complex and not fully elucidated process. It is currently understood that UC predominantly affects individuals with genetic susceptibility, while factors such as epithelial barrier defects, dysbiosis, and dysregulated immune responses play significant roles in its pathogenesis^3–5^. Epidemiologically, the incidence and prevalence of UC have been a dramatic rise in recent years. Globally, the highest incidence and prevalence are in Northern Europe, 505 per 100,000 in Norway, followed by North America, 286 per 100,000 in the USA^6^. The annual incidence of UC in Europe has surged to 24.3 cases per 100,000 individuals, and there is a clear upward trajectory in both the prevalence and incidence of UC over time^7^. It’s worth noting that in many emerging industrialized countries in South America, Asia, and Africa, although the prevalence is still low, the number of new UC diagnoses is increasing, and the prevalence is expected to rise in the future^8^. This presents a substantial challenge for healthcare systems on a global scale.

A potential pathogenesis of UC could be immune system dysfunction. When the immune system works hard to resist invading viruses or bacteria, an abnormal immune response can cause the immune system to also attack cells in the digestive tract, leading to chronic intestinal inflammation or mucosal damage. Genetics also play a role as UC is more common in people with family members who have the disease^9^. In the past, UC was commonly managed with 5-aminosalicylates, steroids, and thiopurines. However, despite these treatment options, UC continues to significantly affect patients' quality of life and is associated with a high morbidity rate^10^. Procedures such as ileo-pouch-anal anastomosis and colectomy come with the potential risks of infertility, compromised pouch function, and the development of capsulitis^11^. In recent years, targeted therapeutic agents like tumor necrosis factor (TNF) inhibitors and interleukin inhibitors have garnered increased attention in clinical practice. With ongoing advancements in drug development, there has been a substantial decrease in UC-related mortality, enhancing the overall prognosis for patients with UC^12^. Nonetheless, there is undeniably substantial room for enhancement in the management of UC, as indicated by existing studies that report remission rates (Based on clinical improvements in stool frequency, rectal bleeding, and mucosal appearance on endoscopy, Mayo score) typically falling below 20–30%^12^.

The diagnosis of UC primarily rests on a combination of clinical symptoms, endoscopic findings, histological examination, and exclusion of other causes of colitis, such as infections^13,14^. Serological markers and fecal calprotectin can assist in differentiating UC from other gastrointestinal disorders, but they are not definitive. Looking ahead, there is growing interest in the realm of genetics for diagnostic insights. Recent advancements in genome-wide association studies (GWAS) have identified numerous genetic loci associated with UC susceptibility^15,16^. The clinical symptoms might also correlate with genetic alterations, gene expression profiles in symptomatic controls, from whom inflammatory bowel disease (IBD) had been excluded, resembled those of IBD patients and diverged from healthy controls. The gene expression signatures of these IBD-excluded samples were related to their symptomatic status^17^. Crooke et al. detected the transcript levels of a total of 45 genes in blood by quantitative real-time polymerase chain reaction, and then used ratio score and support vector machine methods to distinguish UC from several types of gastro-intestinal diseases^18^. Recent years, next-generation sequencing is widely applied in disease diagnostic and precision treatment^19,20^. As our understanding of the genetic architecture of UC deepens, it is anticipated that genetic markers could serve as adjunct diagnostic tools, offering more precise disease categorization and personalized therapeutic strategies. This burgeoning area of research holds the promise of reshaping the diagnostic landscape of UC in the future.

The objective of current study is to explore the potential of gene expression profiles in enhancing the accuracy and early detection of UC, particularly in cases where traditional diagnostic methods may be inconclusive. While traditional diagnostics are indeed effective and cost-efficient, gene expression profiling offers several distinct advantages. These include the ability to identify molecular changes at an early stage, which may precede clinical symptoms, thus enabling earlier intervention and potentially improving patient outcomes. In this study, we incorporated soft tissue sequencing data from a cohort of 259 UC patients and 60 individuals without the condition. From this dataset, we identified six key genes and developed a predictive model with a high degree of accuracy for UC diagnosis.

## Methods

### Patients’ summary

We collected a total of eight cohorts contains both health controls and UC patients for the current study. The training datasets derived from mucosal tissue samples included GSE87466 with 21 normal and 87 UC patients, GSE59071 with 11 normal and 97 UC patients, GSE47908 with 15 normal and 45 UC patients, and GSE38713 with 13 normal and 30 UC patients. For validation, the mucosal tissue cohorts comprised GSE53306, which had 12 normal controls, 16 patients in the active UC category and 12 in the inactive UC category. Similarly, GSE13367 had 8 inflamed and 9 non-inflamed UC patients, compared with 10 controls. GSE48958 also from mucosal tissue had 7 active UC and 6 inactive UC patients, accompany with 8 controls. Finally, the GSE126124 dataset, derived from peripheral whole blood, included 39 normal and 18 UC patients (Table 1).

**Table 1:** Basic information of enrolled clinical cohorts.

Type	Tissue	Dataset	Platform	Normal	UC		Source
Training	Mucosa	GSE87466	GPL13158	21	87		https://www.ncbi.nlm.nih.gov/geo/query/acc.cgi?acc=GSE87466
Training	Mucosa	GSE59071	GPL6244	11	97		https://www.ncbi.nlm.nih.gov/geo/query/acc.cgi?acc=GSE59071
Training	Mucosa	GSE47908	GPL570	15	45		https://www.ncbi.nlm.nih.gov/geo/query/acc.cgi?acc=GSE47908
Training	Mucosa	GSE38713	GPL570	13	30		https://www.ncbi.nlm.nih.gov/geo/query/acc.cgi?acc=GSE38713
Validation	Mucosa	GSE53306	GPL14951	12	Active	16	https://www.ncbi.nlm.nih.gov/geo/query/acc.cgi?acc=GSE53306
					Inactive	12	
Validation	Mucosa	GSE13367	GPL570	10	Inflamed	8	https://www.ncbi.nlm.nih.gov/geo/query/acc.cgi?acc=GSE13367
					non-inflamed	9	
Validation	Mucosa	GSE48958	GPL6244	8	Active	7	https://www.ncbi.nlm.nih.gov/geo/query/acc.cgi?acc=GSE48958
					Inactive	6	
Validation	Peripheral whole blood	GSE126124	GPL6244	39	18		https://www.ncbi.nlm.nih.gov/geo/query/acc.cgi?acc=GSE126124

### Mitigating batch effects

Batch effects represent the non-biological discrepancies observed across multiple datasets. To ensure analytical consistency and mitigate biases introduced by such effects, we employed the ComBat algorithms from the "sva" package. This methodology was instrumental in harmonizing the transcriptional profiles of the training cohorts (GSE87466, GSE59071, GSE47908, GSE38713), thus effectively offsetting the intrinsic batch differences among them. For the validation cohort, we abstained from this procedure, as our intent was to further authenticate the diagnostic across diverse platforms.

### Calculation of the scores of signaling pathways

Gene Set Enrichment Analysis (GSEA) is a computational approach ascertaining whether a designated gene set exhibits statistically significant deviations between two groups. We implemented GSEA to initially contrast the various activated signaling pathways between UC patients and healthy controls. The backdrop file of molecular signature gene sets was procured from MSigDB, C5: Biological Process, comprising a total of 7530 gene sets^21,22^.

### An integrative diagnostic model leveraging machine learning techniques

To craft a unified model possessing robust accuracy and stability in distinguishing between UC patients and healthy individuals, we amalgamated 10 machine learning algorithms, yielding 101 algorithmic combinations. The ensemble of algorithms comprised Elastic Net (Enet), Lasso, Ridge, Stepglm[both], Stepglm[backward], glmBoost, Latent Dirichlet Allocation (LDA), NaiveBayes, plsRglm, Random Forest (RF), and Support Vector Machine (SVM). The signature derivation protocol entailed: (1) Isolating the most prominently activated pathways in UC patients across the four GEO cohorts; (2) Subsequently, the 101 algorithmic combinations were executed on the genes curated from these prominently activated pathways; (3) All models underwent training within the GSE55235 dataset and validation in the remaining three cohorts, which remained untouched during pathway filtration; (4) For every model, the AUC metric was ascertained across all participating cohorts.

### Evaluation immunocytes infiltration

Through single-sample gene set enrichment analysis (ssGSEA), the infiltration of immune cells was discerned and evaluated using transcriptional data. The gene collections representing 28 immune cell types were sourced from the research undertaken by Charoentong et al^23^.

### Statistics

Tatistical analyses were conducted using R (Version: 4.2.2). For continuous variables, the Student's t-test and the two-sample Mann–Whitney test were employed for comparisons between two groups if data exhibited a normal distribution, whereas the Wilson rank test was invoked otherwise. A Pearson correlation analysis was employed for continuous datasets. Pertinent pathways were delineated using a heatmap, facilitated by the R package "pheatmap". The Kappa Statistic serves as a metric for contrasting predictive versus actual subtypes. For comparisons across more than two groups, the Kruskal–Wallis test was utilized, and for pairwise assessments, the Wilcoxon test was applied^24^. A two-tailed P value below 0.05 was considered to indicate statistical significance.

## Results

### Summarize of the process

**Figure 1:**
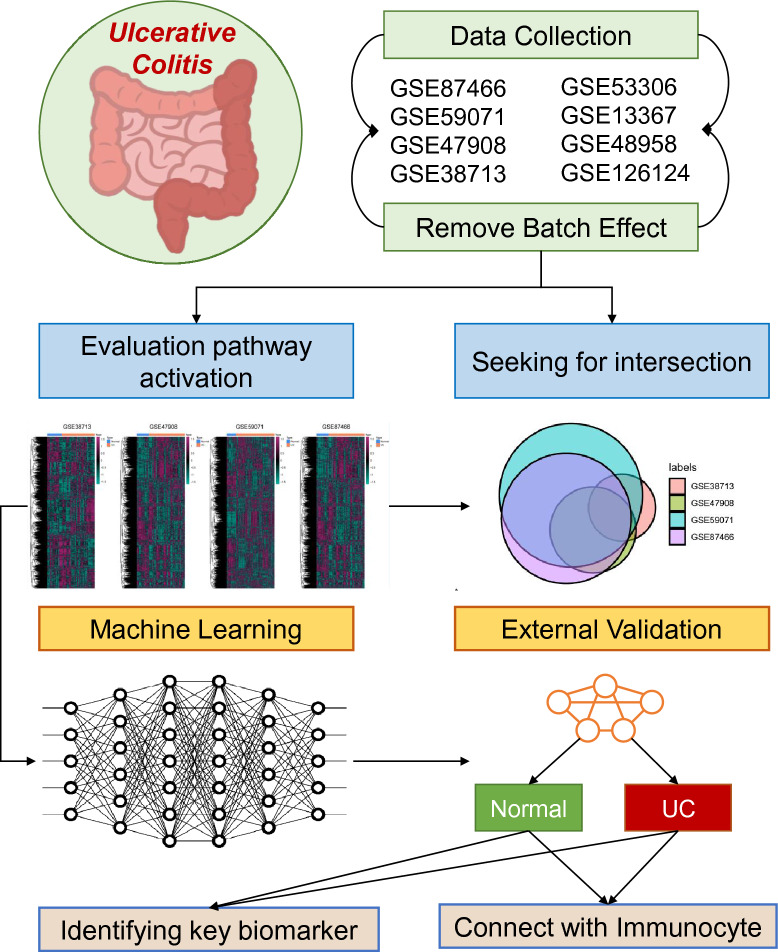
Flowchart illustrating the step-by-step methodology of the current study. Starting from transcriptomic data acquisition from four cohorts, through data preprocessing, gene set enrichment analysis, machine learning diagnostics, and concluding with the identification of characteristic genes and their association with immune cell infiltration.

In this study, transcriptomic data from four cohorts, encompassing Ulcerative Colitis (UC) patients and healthy controls, were evaluated to identify key signaling pathways associated with UC. The gene expression profiles underwent batch correction to ensure uniformity and mitigate batch effects. Using Gene Set Enrichment Analysis (GSEA), over 7500 gene sets were computed, each representing a unique cellular signaling pathway. Machine learning techniques were then employed, with the LASSO regression model emerging as the most efficient diagnostic tool with an average AUC value of 0.942. The robustness of this model was validated using external cohorts. From the diagnostic model, 13 characteristic genes were identified and assessed for their expression differences. Three of these genes, LCN2, ASS1, and IRAK3, were particularly noteworthy as they exhibited elevated expression in UC patients. The study further examined the relationship between these genes and immune cell infiltration, establishing their correlation with activated dendritic cells. These findings reinforce the role of immune system dysregulation in UC and introduce potential biomarkers for diagnostic and therapeutic applications. The flowchart of the current study is displayed in Fig. 1.

### Identifying key signaling pathways reflecting UC

**Figure 2:**
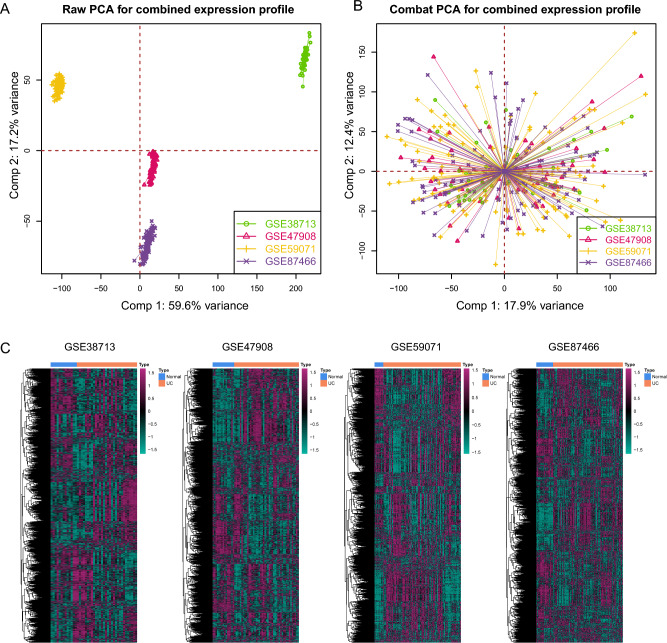
Batch correction and gene set enrichment analysis outcomes. (**A**) Principal component analysis (PCA) plot showing gene expression disparities among the four cohorts prior to batch correction. (**B**) PCA plot post batch correction showcasing uniform gene expression distribution across all cohorts. (**C**) Distribution of scores across 7530 signaling pathways, based on Gene Set Enrichment Analysis (GSEA), for samples in the different cohorts.

As delineated in the methods section, our study incorporated samples from four cohorts, encompassing both UC patients and healthy controls. To ensure uniformity of the transcriptomic data before further analysis, we initially subjected the gene expression profiles from all four cohorts to batch correction. Prior to this correction, the PCA plot exhibited pronounced disparities among the four cohorts (Fig. 2A). However, post-correction, batch effect variations in gene expression distribution across all cohorts were effectively nullified (Fig. 2B). Subsequently, employing the GSEA method, we computed 7530 gene sets, each reflecting the activation status of distinct cellular signaling pathways; each sample included in the analysis garnered a score across these 7530 pathways. The distribution of scores for these pathways across samples in the different cohorts is illustrated in Fig. 2C.

**Figure 3:**
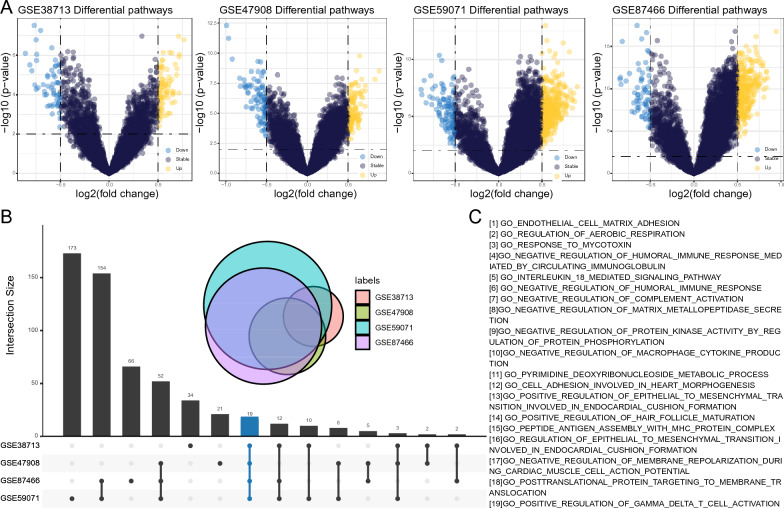
Differentially activated signaling pathways in Ulcerative Colitis (UC) patients versus healthy controls for each cohort. (**A**) Visualization of pathways upregulated in UC patients across the four cohorts. (**B**) Venn diagram illustrating the 19 common upregulated pathways identified across all cohorts. (**C**) List of the names of the 19 upregulated pathways.

Subsequent to this, within each cohort, we discerned signaling pathways that were differentially activated between UC patients and healthy controls (Fig. 3A). In the GSE38713 cohort, 79 pathways were upregulated in UC patients; in the GSE47908 cohort, 428 pathways were upregulated; in the GSE59071 cohort, 107 pathways were upregulated, and in the GSE87466 cohort, 3,609 pathways saw upregulation in UC patients. By extracting the intersecting upregulated pathways across the four cohorts, a total of 19 pathways were finalized (Fig. 3B). These 19 pathways pertained to cell adhesion and development, cell respiration and metabolism, immune response and signaling, as well as regulation of protein activity and secretion (Fig. 3C). Excluding the redundant genes within these pathways, a total of 83 unique genes remained.

### Machine learning constructs a model for identifying patients with UC

**Figure 4:**
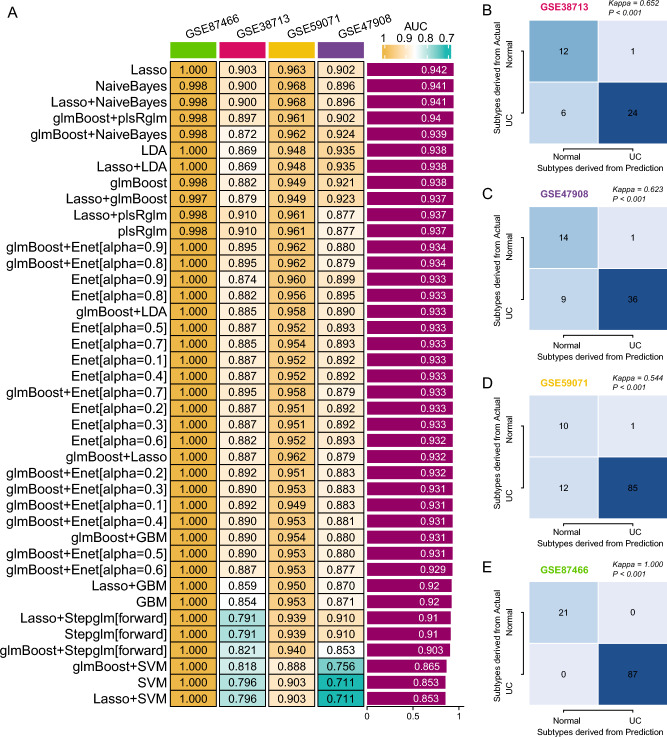
Machine learning-based diagnostic model evaluation. (**A**) AUC values of the 40 types of machine learning model across the four cohorts. (**B**–**E**) Kappa statistics for GSE38713 (**B**), GSE47908 (**C**), GSE59071 (**D**), and GSE87466 (**E**) comparing predicted outcomes with actual UC statuses.

The predictors used as input for the ML models are the gene expression levels of the 83 identified genes. These variables are continuous, representing the expression levels of each gene. Through the iterative analysis of the selected 83 genes across 101 algorithm combinations, 40 combination models were successfully generated. These models displayed their predictive capabilities across different cohorts using AUC values, with the average AUC value across four cohorts also being computed (Fig. 4A). Ultimately, the LASSO regression model demonstrated superior diagnostic capabilities (Average AUC = 0.942). The prediction score can be calculated with the formula: Score = 0.03328012 × SYK + 0.51625614 × CALR − 0.14331840 × GATA5 + 1.29808010 × FLRT2 + 0.80143919 × IRAK3 − 0.59448664 × DUSP26 + 0.85254969 × SPINK5 + 0.25364614 × PTPN6 + 0.44029637 × LCN2 + 0.70178103 × ASS1 + 0.20803807 × BAK1 + 0.70268334 × VCP + 0.27895531 × ACTN3.

Based on the LASSO model, the AUC values for the GSE87466, GSE38713, GSE59071, and GSE47908 cohorts were 1, 0.903, 0.963, and 0.902, respectively. Further, the Kappa statistic was employed to evaluate the heterogeneity between predicted and actual outcomes, revealing that the novel diagnostic model exhibited robust predictive power across all four cohorts (GSE87466: Kappa = 1, P < 0.001; GSE38713: Kappa = 0.652, P < 0.001; GSE59071: Kappa = 0.544, P < 0.001; GSE47908: Kappa = 0.623, P < 0.001; Fig. 4B–E).

### Verifying the efficacy of the diagnostic model in external cohorts

**Figure 5:**
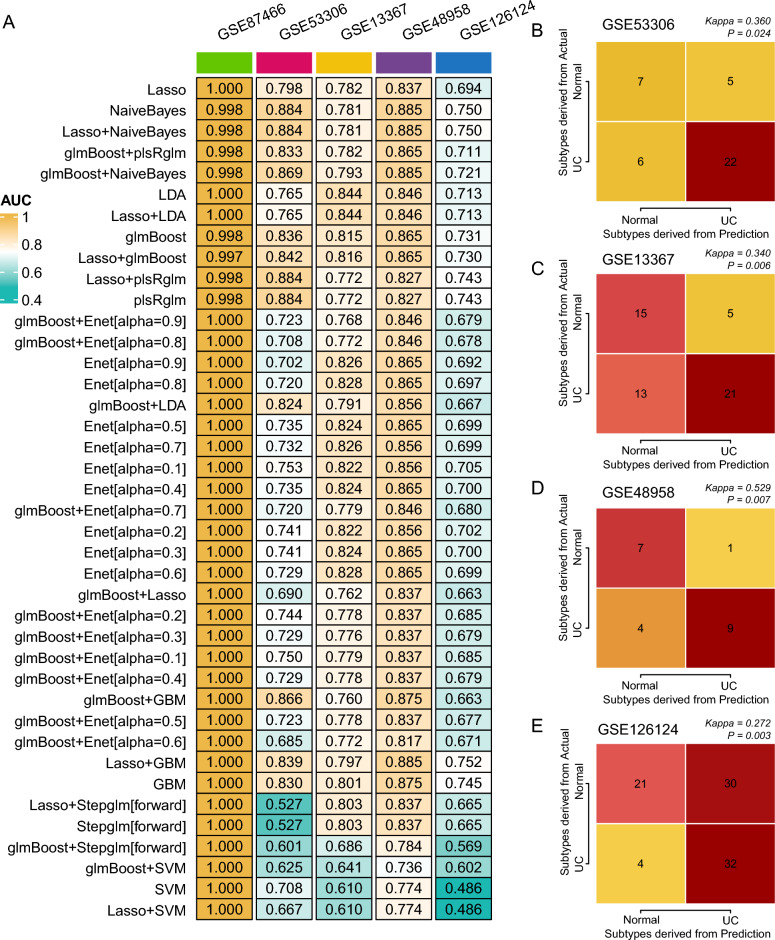
Assessing of the LASSO-based diagnostic model on external cohorts. (**A**) Overall diagnostic performance across the four external cohorts. (**B**–**E**) Detailed diagnostic metrics including AUC, Kappa, and P-values for external cohort, GSE53306 (**B**), GSE13367 (**C**), GSE48958 (**D**), and GSE126124 (**E**).

To further ascertain the diagnostic capabilities of the model, we included four external cohorts: GSE53306, GSE13367, GSE48958, and GSE126124. The samples from the first three cohorts were derived from intestinal mucosal tissue, while the GSE126124 cohort utilized peripheral blood samples from patients and healthy controls. Using the same methodology, we computed the predictive results of the four external cohorts across the 40 models. Ultimately, the LASSO-based diagnostic model consistently showcased commendable diagnostic prowess (Fig. 5A) with the following results: GSE53306 (AUC = 0.798, Kappa = 0.360, P = 0.024, Fig. 5B), GSE13367 (AUC = 0.782, Kappa = 0.340, P = 0.006, Fig. 5C), GSE48958 (AUC = 0.873, Kappa = 0.529, P = 0.007, Fig. 5D). For the GSE126124 cohort, although the AUC value was only 0.694, considering that these samples were derived from peripheral blood, its predictive capability near 0.7 remains a valuable asset for clinical diagnosis (Kappa = 0.272, P = 0.003, Fig. 5E).

### Expression of 13 characteristic genes in UC

**Figure 6:**
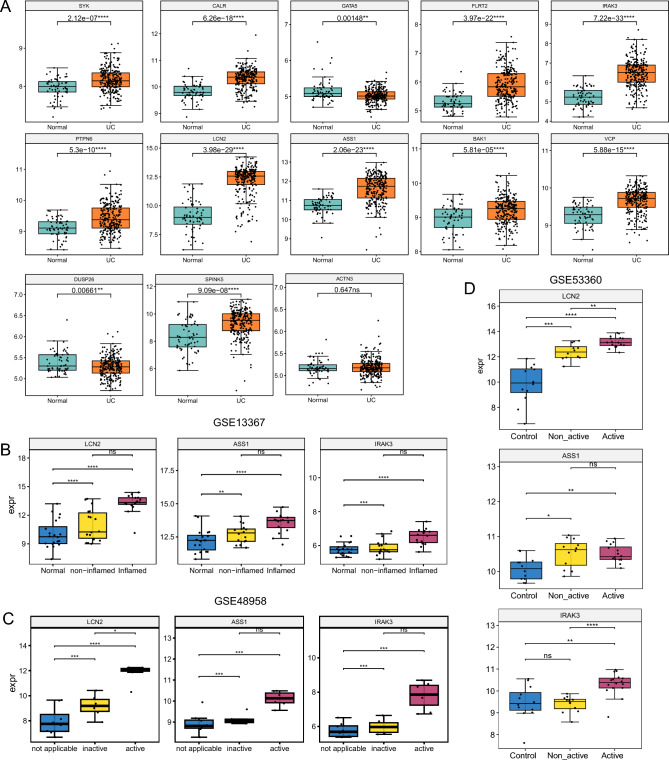
Expression profiles of the 13 characteristic genes. (**A**) Expression differences between UC patients and healthy controls for the identified genes in a merged training cohort. (**B**–**D**) Validation of expression patterns of LCN2, ASS1, and IRAK3 in three external datasets, GSE13367 (**B**), GSE48958 (**C**), and GSE53360 (**D**).

The LASSO logistic regression analysis incorporated 13 genes into the model, namely SYK, CALR, GATA5, FLRT2, IRAK3, DUSP26, SPINK5, PTPN6, LCN2, ASS1, BAK1, VCP, and ACTN3. To elucidate the conditions of these 13 genes, their expression differences between UC patients and healthy controls in a training cohort amalgamated from four cohorts were initially assessed. Notably, 11 out of these 13 genes exhibited significantly heightened expression in UC patients, while DUSP26 manifested diminished expression and ACTN3 showcased no significant difference (Fig. 6A). We selected three significantly upregulated genes in UC, namely LCN2, ASS1, and IRAK3, for further validation in external cohorts. In the GSE13367 dataset, the expression of three genes was notably elevated in UC patients compared to healthy controls. Although these genes exhibited higher expression in inflamed UC patients, there was no statistically significant difference when compared to non-inflamed patients (Fig. 6B). In the GSE48958 dataset, the expression trends of these genes mirrored the previously described patterns, with LCN2 showing the highest expression in active UC patients (Fig. 6C). In the GSE53360 dataset, we observed that LCN2 also had the highest expression in active UC patients, with significant differences when compared both to non-active patients (P < 0.05) and to healthy controls (P < 0.05) (Fig. 6D). These findings indicate that LCN2, ASS1, and IRAK3 are crucial markers distinguishing between healthy controls and UC patients.

### Correlation between key biomarkers and immune cell infiltration

**Figure 7:**
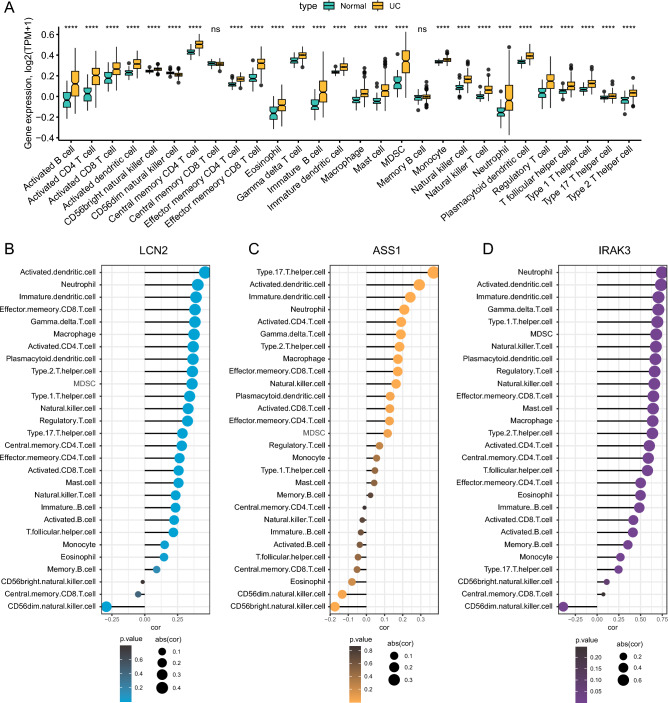
Analysis of immune cell infiltration in UC and its relationship with LCN2, ASS1, and IRAK3. (**A**) Differences in immune cell distribution between UC patients and normal controls. (**B**–**D**) Correlation plots showcasing associations between LCN2 (**B**), ASS1 (**C**), and IRAK3 (**D**) and various immune cells.

A plethora of research concurs that immune system dysregulation is a critical factor precipitating the onset of UC. Consequently, a comparison was made between all included normal controls and UC patients to discern differences in immune cell distribution. It was discerned that the majority of immune cells exhibited pronounced expression elevation in UC patients, most notably myeloid-derived suppressor cell (MDSC), Neutrophil, and central memory CD4 T cells (Fig. 7A). Subsequent investigations evaluated the relationship between LCN2, ASS1, IRAK3, and immune cell infiltration in all UC patients. All three genes exhibited positive correlations with the majority of immune cells, with the strongest associations found with activated dendritic cells, neutrophils, and immature dendritic cells (Fig. 7B–D). Additionally, correlations were established between LCN2 and Effector memory CD8 T cells as well as Gamma delta T cells (Fig. 7B); ASS1 and Type 17T helper cells (Fig. 7C); and IRAK3 with Type 1T helper cells and Gamma delta T cells (Fig. 7D).

**Figure 8:**
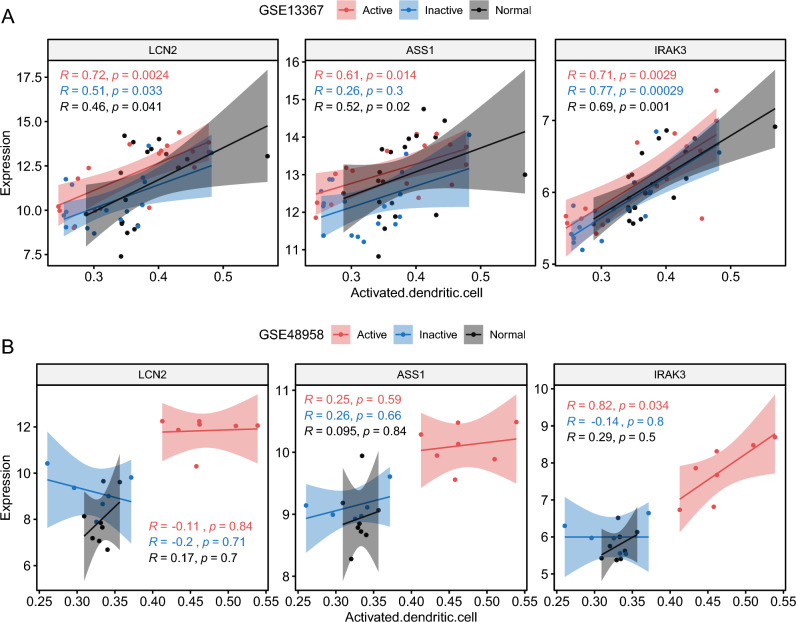
Correlation of LCN2, ASS1, and IRAK3 with activated dendritic cells across different UC disease statuses. (**A**) Correlations in the GSE13367 cohort for three genes and UC patients. (**B**) Correlation in the GSE48958 cohort for three genes and UC patients.

It was observed that all three genes had a pronounced positive correlation with activated dendritic cells. Therefore, further analysis delved into the relationship between these genes and different UC disease statuses. In the GSE13367 cohort, the strongest correlations in active UC patients with activated dendritic cells were noted (LCN2: R = 0.72, P = 0.0024; ASS1: R = 0.61, P = 0.014; IRAK3: R = 0.71, P = 0.0029; Fig. 8A). In the GSE48958 cohort, only IRAK3 exhibited a positive correlation with activated dendritic cells in active UC patients (R = 0.82, P = 0.034, Fig. 8B).

## Discussion

UC remains a focal point in gastroenterological research due to its multifaceted etiological profile and the intricacies associated with its management^25,26^. Developing a robust diagnostic model that can accurately differentiate between UC patients and healthy individuals could offer a paradigm shift in the management of this condition. The application of machine learning in biomedical research has surged exponentially in recent years, with its prowess in data handling and pattern recognition being especially transformative for complex datasets^27–29^. The present study exemplifies this paradigm shift by utilizing machine learning to sift through intricate gene expression profiles, leading to the elucidation of a diagnostic model for UC.

In the current study, four training cohorts were utilized to identify key pathways and genes, leading to the construction of the prediction model in GSE87466, followed by internal validation and subsequent external validation. GSE87466, comprising the largest sample size, was selected for model construction. We did not amalgamate all four training cohorts into a single extensive dataset due to the potential substantial batch effects within the cohorts. For the external validation cohort, GSE126124 comprises samples from peripheral whole blood, whereas the training cohort GSE87466 includes samples from mucosa. In summary, this study encompasses training, internal validation, external validation, and further validation with peripheral whole blood samples to ensure the diagnostic model's robustness and credibility. Central to our findings is the delineation of specific cellular pathways and genes that are distinctly altered in UC patients. Notably, the pathways identified in our study encompass a broad spectrum of cellular processes, ranging from cell adhesion to immune signaling, reinforcing the notion of UC as a systemic ailment with widespread cellular repercussions^30,31^. In subsequently study, the iterative analysis of 83 genes across 101 algorithm combinations is testament to this capability. It is noteworthy that out of these numerous combinations, a set of 40 viable diagnostic models emerged, showcasing the flexibility and rigor of machine learning in generating a suite of models tailored to the data's nuances. The Average AUC value of 0.942 achieved by the LASSO model, and its robust predictive power across all four cohorts, underscore its efficacy. In addition, the model demonstrating remarkable diagnostic precision across multiple external validation cohorts. The strength of the model, as evidenced by its high average AUC value, suggests that gene expression profiling can serve as a formidable tool in the diagnostic arsenal against UC. Furthermore, the robustness of this model, even when applied to peripheral blood samples, underscores its potential versatility and broad applicability in clinical settings.

The incorporation of machine learning also allowed for the identification of 13 key genes, which upon further validation, revealed LCN2, ASS1, and IRAK3 as pivotal markers distinguishing between healthy individuals and UC patients. It is well-established that UC is characterized by chronic inflammation of the colon, predominantly driven by an aberrant immune response^32^. In this study, the robust correlation observed between the expression levels of LCN2, ASS1, and IRAK3 and specific immune cell populations, particularly activated dendritic cells, highlights the intertwined relationship between these genes and immune cell activity in UC^33^. Dendritic cells are known to play a pivotal role in antigen presentation and initiation of adaptive immune responses, their activation could subsequently lead to the recruitment and activation of other immune cells, perpetuating the inflammatory cascade observed in UC^34,35^. Notably, LCN2 has been previously documented to play a role in innate immunity, being associated with neutrophil function and acting as a bacteriostatic agent by sequestering iron, which in turn limits bacterial growth^36–38^. Although we observed that IRAK3 is correlated with the infiltration of activated dendritic cell, however, it can not distinguish the disease status of UC, the potential reason is that in the UC cases, inflammation and tissue remodeling of uninflamed (inactive) regions similar to inflamed (active) regions, they all have the increased expression of TGF -β, vimentin, and α-SMA^39^.

Combining various methods in a multi-faceted research setup presents a range of benefits and drawbacks. One significant advantage is the increased robustness and reliability of the results. By integrating different techniques, such as machine learning algorithms and gene expression analyses, researchers can cross-validate findings, reducing the likelihood of false positives and enhancing the overall confidence in the results. Additionally, the flexibility in combining methods can facilitate the discovery of novel biomarkers and therapeutic targets, providing a holistic view of disease mechanisms and potential intervention points. However, there are inherent drawbacks to this approach. The complexity of managing and integrating diverse datasets and methodologies can be challenging, requiring advanced computational skills and substantial computational resources. The risk of overfitting increases with the use of multiple machine learning models, where a model may perform exceptionally well on training data but poorly on unseen data, thus limiting its generalizability. Furthermore, while combining methods can highlight potential biomarkers or pathways, it often does not provide mechanistic insights into their roles, necessitating further functional studies to elucidate their contributions to disease pathogenesis. Therefore, while the integration of multiple methods can significantly advance our understanding and management of diseases like UC, it requires careful consideration of these potential limitations.

While the advantages of machine learning are manifold, it is vital to approach its results with a measure of caution, and there are several limitations for the current study. First, this study utilized a relatively small cohort of patients. Larger and more varied cohorts are necessary to validate the diagnostic model across different demographic groups. Second, the external validation cohorts primarily consisted of mucosal tissue samples, with only one cohort (GSE126124) derived from peripheral blood. The diagnostic model's performance in blood samples was lower (AUC = 0.694) compared to mucosal samples, indicating the need for further refinement and validation in non-invasive sample types like blood. Third, while the study identified several key genes and pathways associated with UC, it did not provide detailed mechanistic insights into how these genes contribute to the disease's pathogenesis. Functional studies are necessary to elucidate the biological roles of these genes and their potential as therapeutic targets.

## Conclusion

In conclusion, our research epitomizes the transformative potential of machine learning in the realm of UC research, offering hope for more accurate and early diagnosis. As we stand on the cusp of a new era in personalized medicine, integrating machine learning insights with traditional biomedical research could pave the way for novel therapeutic avenues and improved patient outcomes. Future studies should prioritize external validation of these models in diverse populations and delve deeper into the functional roles of identified biomarkers.

## Data Availability

All the datasets presented in this study can be obtained from the GEO (http://www.ncbi.nlm.nih.gov/geo) database, and details listed in Table 1. Data is provided within the manuscript or supplementary information files and it is available upon request from the corresponding author.
